# Enhanced Separation Performance of Polyamide Thin-Film Nanocomposite Membranes with Interlayer by Constructed Two-Dimensional Nanomaterials: A Critical Review

**DOI:** 10.3390/membranes12121250

**Published:** 2022-12-10

**Authors:** Yifei Yu, Xianjuan Zhang, Peng Lu, Dingbin He, Liqiang Shen, Yanshuo Li

**Affiliations:** 1 School of Materials Science and Chemical Engineering, Ningbo University, 818 Fenghua Road, Ningbo 315211, China; 2 Hymater Co., Ltd., 777 Qingfeng Road, Ningbo 315000, China; 3 Ningbo Shuiyi Membrane Technology Development Co., Ltd., 368 Xingci One Road, Ningbo 315336, China

**Keywords:** thin-film, two-dimensional nanomaterials, interlayer, permeability and selectivity, separation membrane

## Abstract

Thin-film composite (TFC) polyamide (PA) membrane has been widely applied in nanofiltration, reverse osmosis, and forward osmosis, including a PA rejection layer by interfacial polymerization on a porous support layer. However, the separation performance of TFC membrane is constrained by the trade-off relationship between permeability and selectivity. Although thin-film nanocomposite (TFN) membrane can enhance the permeability, due to the existence of functionalized nanoparticles in the PA rejection layer, the introduction of nanoparticles leads to the problems of the poor interface compatibility and the nanoparticles agglomeration. These issues often lead to the defect of PA rejection layers and reduction in selectivity. In this review, we summarize a new class of structures of TFN membranes with functionalized interlayers (TFNi), which promises to overcome the problems associated with TFN membranes. Recently, functionalized two-dimensional (2D) nanomaterials have received more attention in the assembly materials of membranes. The reported TFNi membranes with 2D interlayers exhibit the remarkable enhancement on the permeability, due to the shorter transport path by the “gutter mechanism” of 2D interlayers. Meanwhile, the functionalized 2D interlayers can affect the diffusion of two-phase monomers during the interfacial polymerization, resulting in the defect-free and highly crosslinked PA rejection layer. Thus, the 2D interlayers enabled TFNi membranes to potentially overcome the longstanding trade-off between membrane permeability and selectivity. This paper provides a critical review on the emerging 2D nanomaterials as the functionalized interlayers of TFNi membranes. The characteristics, function, modification, and advantages of these 2D interlayers are summarized. Several perspectives are provided in terms of the critical challenges for 2D interlayers, managing the trade-off between permeability, selectivity, and cost. The future research directions of TFNi membranes with 2D interlayers are proposed.

## 1. Introduction

**Figure 1:**
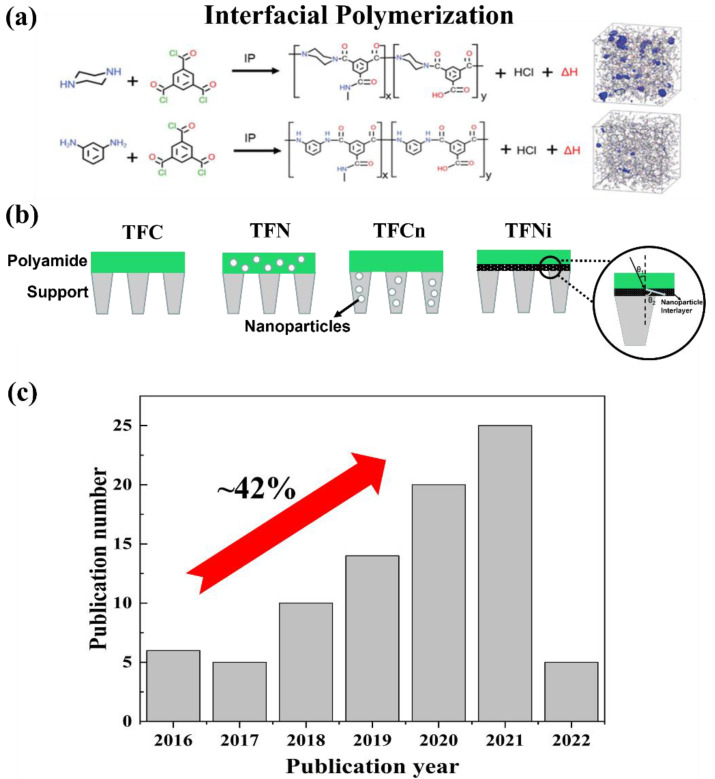
(**a**) Interfacial polymerization reactions between the 1,3-phenylenediamine or piperazine with the 1,3,5-benzenetricarbonyl trichloride, as well as the molecular structure of the corresponding PA layers from molecular dynamics simulations, reproduced with permission from Ref. [48] with copyright permission from © 2020 American Chemical Society; (**b**) schematic illustration of the structures of TFC, TFN, TFCn, and TFNi membranes; (**c**) Recent publications on the TFNi membranes with 2D interlayers. All data were obtained by searching the keyword “TFNi” in the database of Scopus, under May 2022, with further manual screening.

Membrane technology has been rapidly developing in the past 60 years, and it is widely used to remove unwanted contaminants in drinking water, wastewater treatment, and seawater desalination [1]. Polyamide (PA) thin-film composite (TFC) membrane, as the most successful commercialization, has experienced tremendous development since the concept of interfacial polymerization (IP) was first introduced by Mogan [2]. The typical PA-TFC membrane consists of the porous support layers and the PA rejection layer formed by the IP reaction from 1,3-phenylenediamine (MPD) or piperazine (PIP) with the 1,3,5-benzenetricarbonyl trichloride (TMC) (Figure 1a). The state-of-the-art PA-TFC membranes are mainly employed for reverse osmosis (RO), nanofiltration (NF), and forward osmosis (FO) processes with the prominent water permeability and water/salts selectivity. However, the major challenges of the PA-TFC membrane are the trade-off between water permeability and salts rejection, as well as fouling and chlorination, for the large-scale application [3,4,5].

Compared with the PA-TFC membrane, the thin-film nanocomposite (TFN) membrane is generated by adding nanomaterials into the PA layer, which has been intensively explored over the decades [6]. These nanomaterials in the PA layer created the additional water transport paths, which improved the water permeance and accompanied the additional functionalities, e.g., anti-fouling [7,8] and chlorine resistance [9,10]. The first concept construction of TFN membrane (Figure 1b) was proposed by Hoek et al. [11], which embedded the zeolite nanoparticles throughout the PA layer by IP reaction. The zeolites with charged pores play an active role in the water permeance and NaCl rejection under the RO process. Subsequently, a variety of nanomaterials was introduced into the PA layer, e.g., metal-organic framework (MOF) [12,13,14], graphene-based nanomaterials [15], and clay nanomaterials [16]. The influences and mechanisms of different embedded nanomaterials on the TFN membrane’s performance have been investigated [17,18]. Although several studies indicated that the nanomaterials play an important role on the enhancement of water permeance for the TFN membranes (e.g., water permeance enhancement up to 50-200% [19]), the self-aggregation of nanomaterials and poor compatibility between of nanomaterials and PA layer compromised the water/salt selectivity of membranes [18].

The importance of the support layer’s property on the PA-TFC membranes has attracted wide attention [17,20,21]. The structural (e.g., surface pore size distribution and porosity, overall porosity, and roughness) and material properties (e.g., specific functional groups) of support layers should affect the formed PA layer by IP, which would influence the final performance of PA-TFC membranes [17]. Simultaneously, the nanomaterials can be introduced into the support layers to produce TFC membranes with a nanocomposite substrate (TFCn) (Figure 1b) [22,23,24]. For example, the high-porosity nanocomposite polyetherimide support layer, produced by incorporating silica nanoparticles, was reported by Tian et al. [25]. The nanocomposite substrate demonstrated that the final TFCn membranes had higher mechanical strength and lower structural parameters than the conventional TFC membrane. However, the nanomaterials always encounter the issue of aggregation in the polymer matrix, particularly at high loadings (>2 wt%) [26], which will induce the generation of defects in the final membranes [27,28].

Recently, a novel concept of thin-film nanocomposite membrane with an interlayer (TFNi) (Figure 1b) was proposed, which consists the pre-loading of functionalized nanomaterials as an interlayer on the support layer before forming the PA layer [19,29]. The interlayer can effectively provide nanopores as the “gutter” effect and, therefore, enhance the overall transport efficiency of TFNi membranes [30,31]. The TFNi membranes were reported with the dramatically enhanced NF and RO separation performance compared with the TFN and TFCn membranes [19]. The TFNi membranes are expected to break the trade-off between water permeability and water/salt selectivity.

The functionalized nanomaterials should be carefully chosen for optimizing the interlayer. Diversified nanomaterials bring the limitless possibilities for the interlayer of TFNi membranes. Dimensionality is one of the most fundamental material parameters, which not only defines the atomic structure of the nanomaterials but also determines the properties to a significant degree [32,33,34]. Additionally, two-dimensional (2D) nanomaterials with the unique physicochemical properties have become a hot research topic in the membrane’s community [35,36,37,38]. Then, the 2D nanomaterials also have blossomed as promising for a nanomaterial’s interlayer for the TFNi membranes, e.g., graphene oxide (GO) [39], MXene [40], MoS_2_ [41], MOF [42], and COF [43] nanosheets. The 2D nanomaterial’s interlayer of the reported TFNi membranes is generally obtained by coating [44], vacuum filtration [28], layer-by-layer assembly [45], and in-situ growth [46,47] methods. Employment of these 2D nanomaterials, with the specific gallery as the interlayer of TFNi membranes, could be used for creating the additional transfer paths between stacked nanosheets, as well as improving the water permeability and water/salt selectivity.

Although the reported TFNi membranes with 2D interlayer have been rapidly increased during the past five years, as shown in Figure 1c, the 2D nanomaterial’s interlayer for TFNi membranes has not been well summarized yet. Based on the above advantages and scalability of the 2D interlayer, an overview of the current development was proposed on the 2D nanomaterial’s intercalated TFNi membranes. The TFNi membranes with the different 2D nanomaterials’ interlayers were summarized, which focused on their unique physicochemical and molecular sieving behaviors for high-efficiency separation membranes. Applications of prepared TFNi membranes in water and solvent separation systems were emphasized. This review also provided useful guidance on the rational selection of 2D nanomaterials, as well as preparation methods of the 2D interlayer for the development of high-performance TFNi membranes.

## 2. Two-Dimensional Nanomaterial’s Interlayers

The 2D nanomaterials, as interlayers, provide unique prospects on the performance enhancement of TFNi membranes [49,50,51]. The 2D nanomaterials with one or few atoms thickness are appropriate materials for producing the thin and homogenous nanoscale interlayers [52]. The interlayers of TFNi membranes are constructed by stacking 2D nanomaterials, which may generate the nano-scale channels in the nanosheets slit and gallery [42]. These nano-scale channels can increase the transfer and achieved high-efficiency ion/molecular sieving. Furthermore, the certain 2D nanomaterials have promising mechanical properties and chemical stability, allowing for the utilization of organic solvent separation, even in the hostile chemical conditions [53,54]. In addition, the 2D nanomaterial’s interlayers could improve IP reaction and, thus, the formation of better-quality PA layers. In the following sections, we emphasized the representative 2D nanomaterials as the interlayers for the prepared TFNi membranes, as well as the applications of TFNi membrane in water and solvent separation systems.

### 2.1. Graphene Oxide

Graphene oxide (GO), a chemical derivative of 2D graphene, attracted extensive research for developing high performance membranes [39,55]. With a high area-to-thickness ratio and abundant functional groups on the surface, the GO nanosheets can be stacked on top of each other to form large-area thin films or membranes [56,57]. The well-arranged GO nanosheets showed the promising gas and liquid separation performance. Furthermore, the control of microstructures and modifications of GO nanosheets using various methods have further enhanced the GO membrane performances [58]. However, controlling the pores and scaling up the GO membranes remain great challenges.

**Figure 2:**
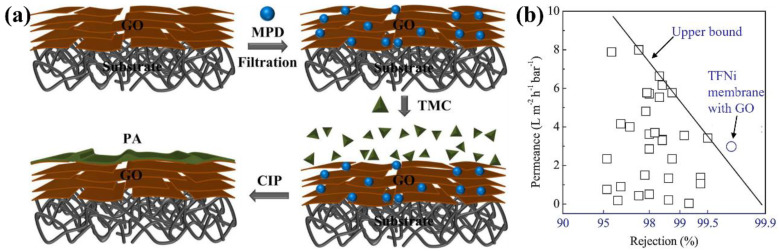
(**a**) Schematic illustration of TFNi membrane with GO nanosheets via confined interfacial polymerization, (**b**) Comparison of the prepared TFNi membrane with other RO membranes (The squares were the published RO membrane, and the circle was TFNi membrane with GO), reproduced with permission from Ref. [59] with copyright permission from © 2018 Elsevier.

The GO nanosheets have received significant attention as promising interlayers. For instance, the GO nanosheets being directly deposited on the surface of support layers [59] and formation of the PA layer on the GO modified support layers were prepared by the IP reaction with MPD and TMC (Figure 2a). The GO nanosheets with oxygen-containing groups could adsorb more amino-contained MPD molecules, which facilitated the controlled formation of PA layers. The results demonstrated that confined IP reaction could fabricate defect-free, highly permeable, and excellently selective RO membranes (Figure 2b). Subsequently, an ultrathin hydrophilic interlayer was proposed for the fabrication of TFNi membranes, which comprised the polydopamine (PDA) modified GO nanosheets by self-polymerization [60]. This hydrophilic interlayer enhanced the pure water permeability of the support layer membranes. The prepared TFNi membrane, with a PDA-modified GO interlayer, showed great improvement of water flux (57.6%) without severe loss of the reverse solute flux under the FO process. In addition, a new technique of plasma-enhanced chemical vapor deposition (PECVD) was proposed for the modification of GO nanosheets. Seah et al. [61] employ the PECVD method to synthesize different types of surface-coated GO nanosheets as interlayers for the fabrication of TFNi membranes using emerging mist-based IP reaction. The effects of hydrophobic/hydrophilic coating on the GO properties and how the changes on its surface chemistry could influence the properties of TFNi membranes for salt/dye mixture treatment were investigated. The prepared TFNi membrane could recover 79-86% of NaCl from the feed, producing saline permeate containing <0.3% pigment.

### 2.2. Mxenes

MXenes are emerging 2D nanomaterials derived from the layered ternary M_n+1_AX_n_, or MAX phases, where ‘M’ is a transition metal, ‘A’ is a group IV-V element, and ‘X’ is carbon and/or nitrogen [62,63]. Currently, more than 30 MXenes nanomaterials have been experimentally synthesized, containing transition metal carbides, nitrides, or carbonitrides [64]. The MXenes with the high surface charge, excellent hydrophilicity, large specific surface area, the changeable molecular structures, and chemical compositions [65], have great potentials in designing and fabricating functional composite membranes.

**Figure 3:**
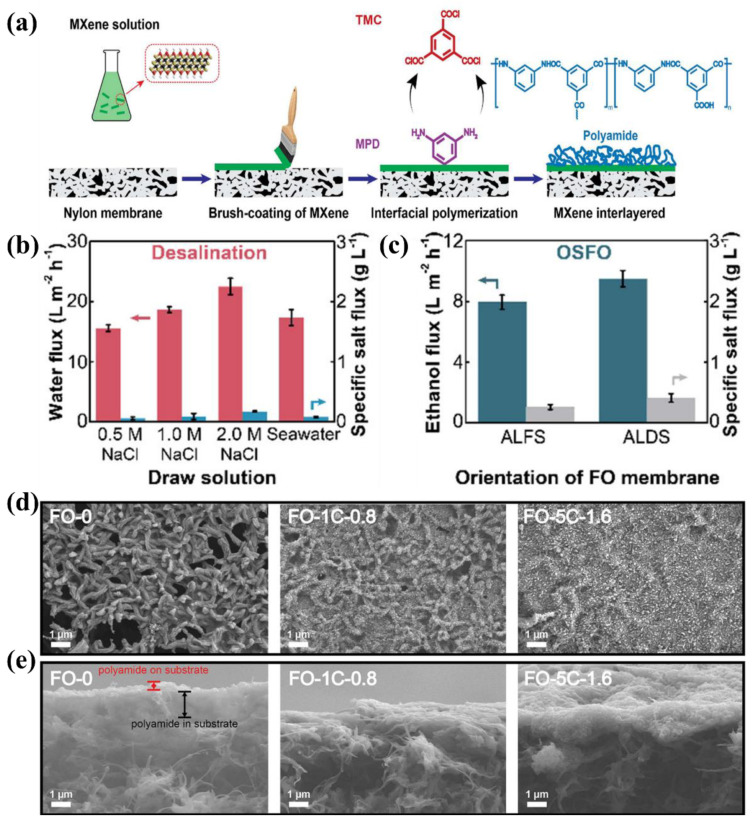
(**a**) Schematic of the fabrication of the TFNi FO membrane with MXene interlayer on the surface of commercial nylon membranes, (**b**) water flux and specific salt flux of the FO-1C-0.8 membrane with different draw solutions in the FO mode, (**c**) ethanol flux and specific salt flux of the FO-1C-0.8 membrane in an organic solvent FO test. (**d**) Surface and (**e**) cross-sectional SEM images of the TFNi FO membrane with MXene interlayer (FO-0 and FO-1C-0.8 membranes, where the numbers represent the coated 0 or 1 times at 0.8 mg L^−1^ concentration of MXene solution), Reproduced with permission from Wu et al. [68], with copyright permission from © 2020 American Chemical Society.

For instance, Ti_3_C_2_T_x_, as a representative of MXenes, is hydrophilic with a negatively charged surface similar to GO nanosheets [66]. The functional groups of grafted Ti_3_C_2_T_x_ nanosheets were employed to fabricate TFN membranes for organic solvent nanofiltration [67]. The 2D MXenes, as the interlayer between the substrates and PA layer, had attracted to improve the performance of FO membranes. Wu et al. [68] used a combination of brush-coating and interfacial polymerization methods (Figure 3a) to prepare the TFNi membranes with Ti_3_C_2_T_x_ nanosheets. The TFNi membrane (FO-1C-0.8) exhibited the outstanding FO desalination performance by increasing the concentration of the draw solutes from 0.5 to 2.0 M (Figure 3b). The average ethanol fluxes of 8.1 and 9.5 L m^−2^ h^−1^ and low reverse solute fluxes of 1.8 and 3.7 g m^−2^ h^−1^ were obtained in the organic solvent FO tests (Figure 3c). These improved results attributed to the adjustment of substrate properties and the PA layer (Figure 3d,e), as well as the facilitation of water and ethanol transportation by the interlayer gallery, between Ti_3_C_2_T_x_ nanosheets. In addition, Sun et al. [40] fabricated a carbon nanotubes (CNT)-intercalated MXene interlayer for a novel TFNi membrane. Compared with the TFC membrane, the prepared TFNi membrane featured an interlayer of the CNT-intercalated MXene, which attained a water flux that was four times higher and a lower specific salt flux under FO tests. Thus, it showed that a highly permeable CNT-intercalated MXene interlayer could provide an enhanced “gutter” effect, and it improved the FO performance of the corresponding TFNi membranes.

### 2.3. Molybdenum Disulfide

**Figure 4:**
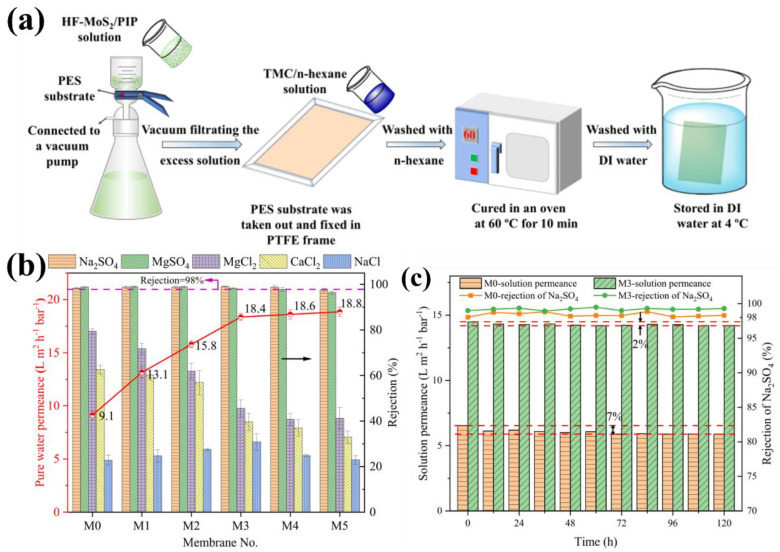
(**a**) The schematic diagram of TFNi membrane fabrication with the MoS_2_ interlayer; (**b**) NF separation performance of prepared TFNi membranes, and the deposition density (0, 2.5, 5.0, 10, 15, and 20 μg/cm^2^) of MoS_2_ nanosheets were denoted as M0, M1, M2, M3, M4, and M5; (**c**) a long-time stability test of TFNi membranes (M0 and M3) within 120 h, reproduced with permission from Wang et al. [72], with copyright permission from © 2022 Elsevier.

Molybdenum disulfide (MoS_2_) is another 2D nanomaterials that has recently attracted greater attention than GO nanosheets [41,69,70]. The MoS_2_ has the three atomic layers, containing the molybdenum atomic layer, sandwiched between two sulfur atomic layers. Then, the MoS_2_ nanosheets show a unique layered morphology that resembled a “sandwich” structure. Simultaneously, the stacked MoS_2_ nanosheets may generate smooth and stiff sub-nanoscale channels, contributing to the high water flux and ion/molecular selectivity [71]. A hierarchical flower-like structure of molybdenum sulfide (HF-MoS_2_) was deposited on the polyethersulfone (PES) ultrafiltration substrate by vacuum filtration [72] (Figure 4a). The excellent hydrophilicity and large specific surface area of HF-MoS_2_ was beneficial to the dispersion of PIP solution by IP reaction; then, a loose and defect-free PA layer was obtained. The results showed that the TFNi membranes with specific density of HF-MoS_2_ had excellent separation performance (e.g., M3 membrane showed the pure water permeance of 18.3 L m^−2^ h^−1^ bar^−1^, and the rejections of Na_2_SO_4_ and MgSO_4_ were above 98%) (Figure 4b). In addition, compared with the TFC membrane (M0), the TFNi membranes (M3) exhibited excellent structural stability in the long-term NF performance evaluation (Figure 4c).

A performance summary of TFNi membranes with the above 2D nanomaterial’s interlayers was shown in Table 1. It was noting that the 2D interlayer intercalated TFNi membranes exhibited the simultaneous enhancement on water/solvent’s fluxes and rejections for RO, NF, and FO processes. In particular, the 2D interlayers were obtained by advanced preparation techniques, e.g., chemical modification and CVD, which showed an enhanced “gutter” effect and improved the separation performance. Then, it was believed that the 2D interlayer may pave a brand-new avenue for designing and fabricating high-performance TFNi membranes.

## 3. Interlayers from Emerging Nanosheets

Metal-organic framework (MOF) and covalent organic framework (COF) are burgeoning porous nanomaterials, which had wide interests in recent years because of their interesting chemistry and potential applications. In membrane science, researchers have extensively explored the use of MOF and COF for gas and liquid separations [75]. For instance, zeolitic-imidazolate framework (ZIF), a subclass of MOF, has been gaining attention in membrane materials for H_2_/CO_2_ separation [76] and ethanol recovery [29]. The ability to exfoliate MOF and COF into ultrathin nanosheets enables a range of new opportunities for the interlayer materials of TFNi membranes.

### 3.1. MOF Nanosheets

MOF is a kind of nano-porous crystalline materials containing the transition metal ions as nodes and organic ligands as connecting chains [77,78]. Exfoliated MOF nanosheets with ultrathin thickness, large surface area, and high surface-to-volume atom ratios are attracting increasing attention in the last few years [48,69,79]. The MOF nanosheets can exploit the voids on the surface of nanosheets to provide a unique diffusion pathway for water or solvent molecules. Meanwhile, the subatomic surface function of MOF nanosheets can effectively enhance the compatibility between PA and MOF, which was beneficial to improve the water/solvent flux and selectivity of TFNi membranes [80]. In this section, we summarize the recent advances in the utilization of MOF nanosheets as interlayer for the prepared TFNi membranes and how they improved the separation performance.

**Figure 5:**
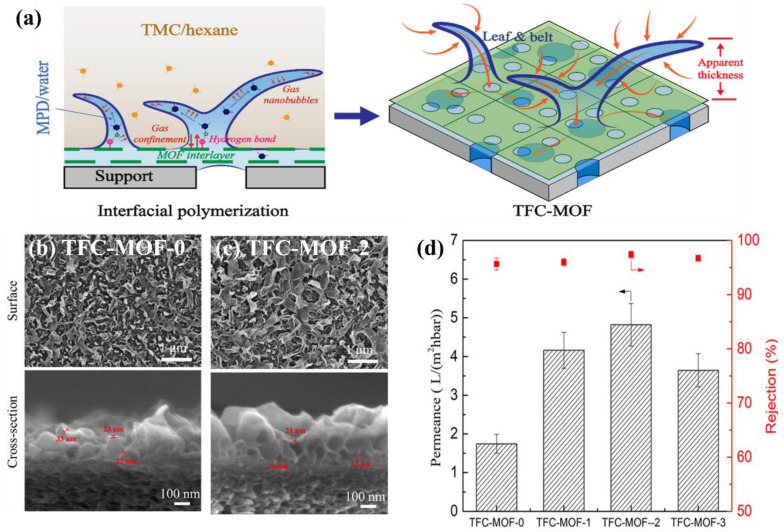
(**a**) Schematic illustration of the effects of incorporating MOF nanosheets on IP process and PA structure; surface and cross-section SEM images of (**b**) TFC-MOF-0 membrane, (**c**) TFC-MOF-2 membrane; (**d**) separation performance of TFC-MOF-0, TFC-MOF-1, TFC-MOF-2, and TFC-MOF-3 membranes, reproduced with permission from Wen et al. [69], with copyright permission from © 2020 American Chemical Society.

To increase water permeability and dye rejections, Carlos et al. [81] created a unique TFNi membrane for the NF process by depositing a ZIF-93 interlayer on the surface of the polyimide (P84) hollow fiber membrane. The ZIF-93 interlayer includes aldehyde functional groups and could be reacted with MPD monomer. This process will neutralize the HCl during interfacial polymerization and form a compact PA layer. Compared with TFC membrane, the TFNi membrane with ZIF-93 interlayer not only improved a four-fold increase in water permeability but also increased the rejection of dye (Sunset yellow, 450 Da) from 88% to 98%. Wen et al. [69] used ultrathin 2D MOF nanosheets (ZnTCPP) as interlayer to fabricate the TFNi membrane (Figure 5a). Compared with the conventional TFC membrane (TFC-MOF-0) (Figure 5b), the larger size and wider leaf-like features of PA layer were observed, and the thinner and defect-free PA layer on the ZnTCPP modified substrates was produced (Figure 5c). Then, the optimal TFNi membrane (TFC-MOF-2) simultaneously increased water permeance (4.82 L m^−2^ h^−1^ bar^−1^) and NaCl rejection (94.7%) under the RO mode test (Figure 5d). Zhao et al. [42] focused on the development of high-performance RO membranes, which used UiO-66-NH_2_ MOF as interlayer to improve the separation efficiency of TFNi membrane. The obtained PA layer on the UiO-66-NH_2_ modified PSf substrate showed a greater surface area, which allowed the formation of additional water transport channels. The prepared TFNi membrane achieved the greater water flux (42 L m^−2^ h^−1^) under the desalination process.

In addition, the TFNi membranes with MOF interlayer have promising applications in an organic solvent system. For instance, Lu’s group [29] demonstrated the facile fabrication of ZIF-8 interlayers for TFNi membranes by simultaneous phase inversion and the crosslinking process. The prepared TFNi membranes with ZIF-8 interlayer had remarkable water and solvent permeances under the RO mode, as well as the higher ethanol flux under organic solvent forward osmosis (OSFO). Zhang et al. [82] successfully synthesized a stable modified ZIF-8 (MZIF-8), as an interlayer on a ceramic support, before interfacial polymerization. The prepared TFNi membranes with MZIF-8 interlayer exhibited higher dehydrating performance for ethanol-water mixtures.

### 3.2. COF Nanosheets

COF is a new class of crystalline organic porous materials, which constructed the periodic networks connected by covalent bonds and are usually referred to as “organic zeolites” [83]. The variety of organic ligand structures may be used to build multifarious COF crystals with 2D structures [84]. Even in strong acid, strong alkali, and hostile solvent conditions, COF offers superior thermal and chemical stability to MOF [43]. The 2D COF nanosheets were prepared by two categories: top-down and bottom-up, which possessed a wide variety of tunable properties [85]. Thus, the 2D COF nanosheets have shown great promising applications in separation [86], energy storage [87], catalysis [88], etc.

Compared with GO nanosheets, the 2D COF nanosheets may serve as ideal nano-interlayers for constructed TFNi membranes, offering additional solvent channels and solute selectivity due to their variable pore size and 2D flat-plate structures [89]. Some competitive fabrication methods, such as in-situ growth and vacuum filtration, have been explored to produce continuous and defect-free 2D COF interlayers [90,91]. However, how to fabricate 2D COF interlayers with structural integrity and stability is still facing challenges.

**Figure 6:**
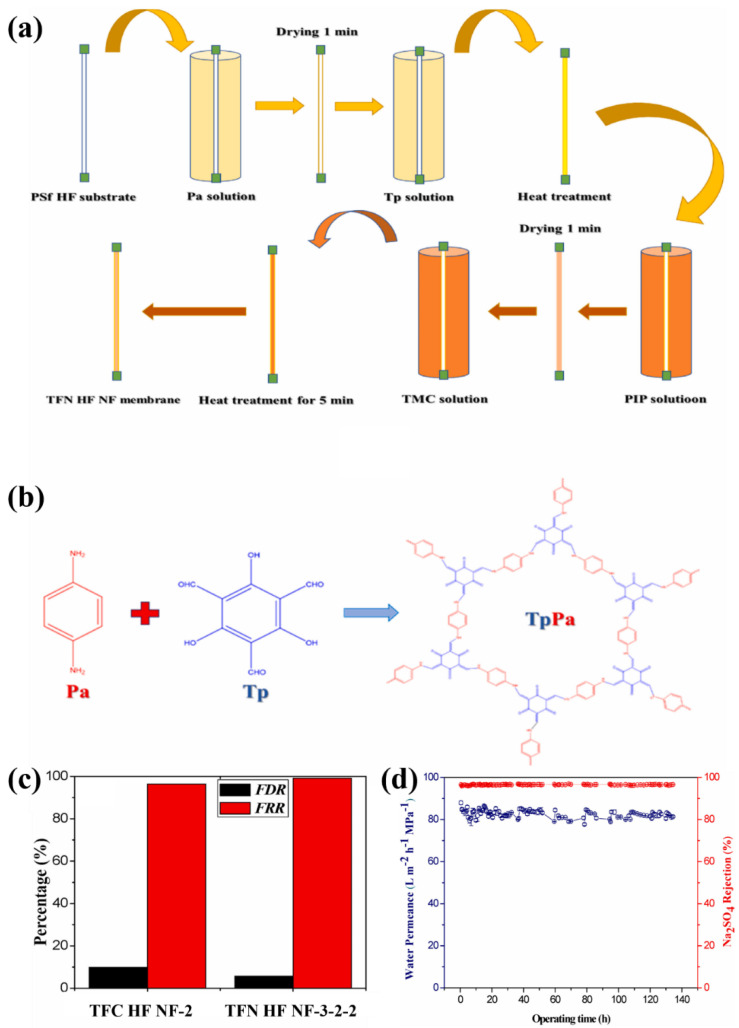
Schematic of (**a**) the IP process for preparing TFN HF NF membrane and (**b**) mechanism of the COF interlayer formation; (**c**) flux reduction rate (FDR) and flux recovery rate (FRR) of TFC and TFNi membranes; (**d**) long-term performance test of TFNi membrane, reproduced with permission from Jiang et al. [91], with copyright permission from © 2021 Elsevier.

Recent studies showed that the uniform deposition of COF nanosheets on the PES support layer, by self-assembly method, can effectively control the interfacial polymerization and reduce the thickness of the PA selective layer (~10 nm). This method reduced the internal concentration polarization (ICP) and improved water transport [90]. The approximately eight-fold enhancement in the water flux of TFNi membrane with COF interlayer was found, without affecting the rejections. Jiang et al. [91] proposed that the COF interlayer enhanced the anti-fouling performance and long-term stability of the final TFNi membrane. They used the direct in-situ growth of COF nanosheets on the PSf hollow fibers (HF) (Figure 6a). The excellent anti-fouling performance (99.1% higher than TFC membrane) was obtained by the BSA fouling experiments (Figure 6c). Meanwhile, the TFNi membranes with COF interlayer exhibited excellent long-term stability (Figure 6d).

He et al. [89] successfully prepared sulfonated COF interlayer on the TMC modified surface of the nylon membrane support by vacuum filtration. The introduction of sulfonated COF interlayer rendered the TFNi membrane with lower water contact angle, more negative charge, thinner PA thickness, and reduced structure parameter. Then, the permeability and selectivity of TFNi membranes were significant enhancements. The prepared membrane overcame the trade-off between the flux and rejection in FO mode, and a superior selectivity (*J_w_/J_s_* = 24.7 L/g) was proposed. In addition, the COF interlayer with structural integrity and stability was beneficial for the preparation of ultra-smooth and ultra-thin PA rejection layers. Yang et al. [43], in-situ, constructed a nano-porous interlayer of 2D COF (TpPa-1) on the PI support for organic solvent nanofiltration (OSN). The introduced COF interlayer interacted with the MPD, leading to an accelerated self-sealing and self-termination of the IP process. The synergetic effect resulted in an ultra-thin PA rejection layer and a greatly increased ethanol permeance. Then, the prepared TFNi membrane showed an ethanol permeance of 60 L m^−2^ h^−1^ MPa^−1^, and the rejection rate of Rhodamine B was higher than 99% (Table 2).

## 4. Conclusions and Perspectives

The TFNi membranes with interlayer have been receiving significant attention in recent years, and most of the current research mainly focuses on water and/or solvent separation processes. The interlayer constructed from 2D nanomaterials has injected new vitality into the TFNi membrane. For instance, the 2D interlayer was facilitated to regulate IP reaction (e.g., distribution of the amine monomer), leading to uniform and dense PA selective layers. The 2D COF nanosheets interlayer reduces the thickness of the PA selective layer by IP reaction. Furthermore, there are significant enhancements on the long-term stability, mechanical strength, chlorine resistance, and anti-fouling capabilities of the final TFNi membranes. In this review, a new generation composite membrane—TFNi with 2D interlayer—was reviewed for water and solvent separation processes. The 2D nanomaterials usually have high aspect ratios and large lateral size; thus, they can easily cover the macropores on the substrate through several layers of stacking, providing a smooth and uniform interlayer needed for IP process. Moreover, the oriented assembly of 2D nanomaterials provides a low friction flow [92], which ensures a low permeation resistance and improves the “gutter” effects.

Nevertheless, the 2D nanomaterials’ interlayers facing major challenges are facile preparation, low-cost, and stability. Based on these considerations, the dip-coating method may be the most practical way for preparation of a 2D interlayer in view of its simple operation, relatively low-cost, and its existing use in commercial membrane production lines. Moreover, the stability of emerging 2D MOF or COF nanosheets’ interlayers is still needed to develop for industrial implementation. Meanwhile, the performance of TFNi membranes strongly depends on microstructures and functionalization of 2D interlayers. Future studies need to perform a more systematic development on the scalable methods for deliberate control of 2D interlayers, especially microstructures and functionalization. More novel and accurate transport models and experiments should be proposed for the unique 2D interlayer in the TFNi membranes. It is significantly important to understand the interaction between PA and 2D interlayer (either physically or chemically), as well as the actual transport mechanism of solutes in the TFNi membranes, particularly those incorporated with nanosheets/nanoporous nanomaterials. Ultimately, the selection and utilization of 2D nanomaterials as interlayers for the fabrication of TFNi membranes should depend on the constituent of feed solutions, e.g., salt ions, dyes, active pharmaceutical ingredients, etc.

## Figures and Tables

**Table 1:** Performances of TFNi membranes with representative 2D nanomaterials interlayers.

2D Nanomaterials	Fabrication Methods	Supports	Flux (L m^−2^ h^−1^)	Selectivity	Ref.
GO	Vacuum filtration	Cellulose	DI-water: 24 (FO: 1 M NaCl draw solution)	NaCl: 99.7%	[59]
PDA/GO	Immersion	PSf	DI-water: 24.296 (FO: 1 M NaCl draw solution)	NaCl: 0.157 g L^−1^	[60]
AA/GO	Vacuum filtration	PSf	DI-water: 90.72 (RO: 8 bar)	Na_2_SO_4_: 95%	[61]
GO	Electro-static atomization	PEI	Acetone: 146.8 (NF: 1 bar)	Brilliant blue: >95%	[73]
PDA/MXene	Surface coating	PES	DI-water: 31 (FO: 1 M NaCl draw solution)	NaCl: 0.28 g L^−1^	[40]
MXene	Brush-coating	Nylon	EtOH: 8.1 (FO: 2 M LiCl draw solution)	LiCl: 0.31 g L^−1^	[68]
PEI/MoS_2_	Surface coating	TiO_2_-CHF	DI-water: 27.6 (NF: 6 bar)	MgCl_2_: 95.5%	[74]

**Table 2:** Performances of 2D MOF and COF nanosheets intercalated TFNi membranes.

Interlayers	Fabrication Methods	Supports	Flux (L m^−2^ h^−1^)	Selectivity	Ref.
ZIF-8	In-situ growth	PI	Ethanol: 2.35 (OSN: 5 bar)	Polystrene 500: 80.3%	[29]
ZnTCPP	Vacuum filtration	PES	Pure water: 77.12 (RO: 16 bar)	NaCl: 97.4%	[69]
ZIF-93	Microfluidic	PI	Pure water: 1.92 (NF: 8 bar)	Sunset yellow: 98%	[81]
UiO-66-NH_2_	Spray coating	PSf	Pure water: 72.2 (RO: 20 bar)	NaCl: 98.91%	[42]
COFs	In-situ growth	PI	Ethanol: 6 (NF: 10 bar)	Rhodamine B: 99%	[43]
SCOFs	Spraying	Nylon	Pure water: 26.7 (FO: 1 M NaCl draw solution)	NaCl: 0.04 g L^−1^	[89]
COFs	Vacuum filtration	PES	Pure water: 107.1 (NF: 2 bar)	Na_2_SO_4_: 94.3%	[90]
COFs	In-situ growth	PSf HF	Pure water: 43.3 (NF: 5 bar)	Na_2_SO_4_: 96.6%	[91]

## References

[1] Zuo K., Wang K., DuChanois R.M., Fang Q., Deemer E.M., Huang X., Xin R., Said I.A., He Z., Feng Y. (2021). Selective membranes in water and wastewater treatment: Role of advanced materials. Mater. Today.

[2] Lau W.J., Ismail A.F., Misdan N., Kassim M.A. (2012). A recent progress in thin film composite membrane: A review. Desalination.

[3] Nazir A., Khan K., Maan A., Zia R., Giorno L., Schroën K. (2019). Membrane separation technology for the recovery of nutraceuticals from food industrial streams. Trends Food Sci. Technol..

[4] Ravanchi M.T., Kaghazchi T., Kargari A. (2009). Application of membrane separation processes in petrochemical industry: A review. Desalination.

[5] Tijing L.D., Dizon J.R.C., Ibrahim I., Nisay A.R.N., Shon H.K., Advincula R.C. (2020). 3D printing for membrane separation, desalination and water treatment. Appl. Mater. Today.

[6] Lau W., Gray S., Matsuura T., Emadzadeh D., Chen J.P., Ismail A. (2015). A review on polyamide thin film nanocomposite (TFN) membranes: History, applications, challenges and approaches. Water Res..

[7] Eghbalazar T., Shakeri A. (2021). High-Performance Thin-Film nanocomposite forward osmosis membranes modified with Poly(dopamine) coated UiO66-(COOH)(2). Sep. Purif. Technol..

[8] Inurria A., Cay-Durgun P., Rice D., Zhang H., Seo D.-K., Lind M.L., Perreault F. (2019). Polyamide thin-film nanocomposite membranes with graphene oxide nanosheets: Balancing membrane performance and fouling propensity. Desalination.

[9] Asempour F., Akbari S., Kanani-Jazi M.H., Atashgar A., Matsuura T., Kruczek B. (2021). Chlorine-resistant TFN RO membranes containing modified poly(amidoamine) dendrimer-functionalized halloysite nanotubes. J. Membr. Sci..

[10] Xue S.-M., Ji C.-H., Xu Z.-L., Tang Y.-J., Li R.-H. (2018). Chlorine resistant TFN nanofiltration membrane incorporated with octadecylamine-grafted GO and fluorine-containing monomer. J. Membr. Sci..

[11] Jeong B.-H., Hoek E.M., Yan Y., Subramani A., Huang X., Hurwitz G., Ghosh A.K., Jawor A. (2007). Interfacial polymerization of thin film nanocomposites: A new concept for reverse osmosis membranes. J. Membr. Sci..

[12] Samsami S., Sarrafzadeh M.-H., Ahmadi A. (2022). Surface modification of thin-film nanocomposite forward osmosis membrane with super-hydrophilic MIL-53 (Al) for doxycycline removal as an emerging contaminant and membrane antifouling property enhancement. Chem. Eng. J..

[13] Wang J., Wang Y., Zhang Y., Uliana A., Zhu J., Liu J., Van der Bruggen B. (2016). Zeolitic Imidazolate Framework/Graphene Oxide Hybrid Nanosheets Functionalized Thin Film Nanocomposite Membrane for Enhanced Antimicrobial Performance. ACS Appl. Mater. Interfaces.

[14] Lee T.H., Oh J.Y., Hong S.P., Lee J.M., Roh S.M., Kim S.H., Park H.B. (2019). ZIF-8 particle size effects on reverse osmosis performance of polyamide thin-film nanocomposite membranes: Importance of particle deposition. J. Membr. Sci..

[15] Ma X.-H., Guo H., Yang Z., Yao Z.-K., Qing W.-H., Chen Y.-L., Xu Z.-L., Tang C.Y. (2019). Carbon nanotubes enhance permeability of ultrathin polyamide rejection layers. J. Membr. Sci..

[16] Dong H., Wu L., Zhang L., Chen H., Gao C. (2015). Clay nanosheets as charged filler materials for high-performance and fouling-resistant thin film nanocomposite membranes. J. Membr. Sci..

[17] Wang L., Kahrizi M., Lu P., Wei Y., Yang H., Yu Y., Wang L., Li Y., Zhao S. (2021). Enhancing water permeability and antifouling performance of thin-film composite membrane by tailoring the support layer. Desalination.

[18] Yin J., Zhu G., Deng B. (2016). Graphene oxide (GO) enhanced polyamide (PA) thin-film nanocomposite (TFN) membrane for water purification. Desalination.

[19] Yang Z., Sun P.-F., Li X., Gan B., Wang L., Song X., Park H.-D., Tang C.Y. (2020). A Critical Review on Thin-Film Nanocomposite Membranes with Interlayered Structure: Mechanisms, Recent Developments, and Environmental Applications. Environ. Sci. Technol..

[20] Li X., Li Q., Fang W., Wang R., Krantz W.B. (2019). Effects of the support on the characteristics and permselectivity of thin film composite membranes. J. Membr. Sci..

[21] Xu G.-R., Xu J.-M., Feng H.-J., Zhao H.-L., Wu S.-B. (2017). Tailoring structures and performance of polyamide thin film composite (PA-TFC) desalination membranes via sublayers adjustment-a review. Desalination.

[22] Lee J., Jang J.H., Chae H.-R., Lee S.H., Lee C.-H., Park P.-K., Won Y.-J., Kim I.-C. (2015). A facile route to enhance the water flux of a thin-film composite reverse osmosis membrane: Incorporating thickness-controlled graphene oxide into a highly porous support layer. J. Mater. Chem. A.

[23] Pendergast M.M., Ghosh A.K., Hoek E. (2013). Separation performance and interfacial properties of nanocomposite reverse osmosis membranes. Desalination.

[24] Park M.J., Lim S., Gonzales R.R., Phuntsho S., Han D.S., Abdel-Wahab A., Adham S., Shon H.K. (2019). Thin-film composite hollow fiber membranes incorporated with graphene oxide in polyethersulfone support layers for enhanced osmotic power density. Desalination.

[25] Tian M., Wang Y.-N., Wang R., Fane A.G. (2017). Synthesis and characterization of thin film nanocomposite forward osmosis membranes supported by silica nanoparticle incorporated nanofibrous substrate. Desalination.

[26] Lu P., Li W., Yang S., Wei Y., Zhang Z., Li Y. (2019). Layered double hydroxides (LDHs) as novel macropore-templates: The importance of porous structures for forward osmosis desalination. J. Membr. Sci..

[27] Fan H., Shan L., Meng H., Zhang G. (2018). High-throughput production of nanodisperse hybrid membranes on various substrates. J. Membr. Sci..

[28] Song X., Zhou Q., Zhang T., Xu H., Wang Z. (2016). Pressure-assisted preparation of graphene oxide quantum dot-incorporated reverse osmosis membranes: Antifouling and chlorine resistance potentials. J. Mater. Chem. A.

[29] Wei Y., Yang Z., Wang L., Yu Y., Yang H., Jin H., Lu P., Wang Y., Wu D., Li Y. (2021). Facile ZIF-8 nanocrystals interlayered solvent-resistant thin-film nanocomposite membranes for enhanced solvent permeance and rejection. J. Membr. Sci..

[30] Yang Z., Wang F., Guo H., Peng L.E., Ma X.-H., Song X.-X., Wang Z., Tang C.Y. (2020). Mechanistic Insights into the Role of Polydopamine Interlayer towards Improved Separation Performance of Polyamide Nanofiltration Membranes. Environ. Sci. Technol..

[31] Zhao W., Liu H., Liu Y., Jian M., Gao L., Wang H., Zhang X. (2018). Thin-Film Nanocomposite Forward-Osmosis Membranes on Hydrophilic Microfiltration Support with an Intermediate Layer of Graphene Oxide and Multiwall Carbon Nanotube. ACS Appl. Mater. Interfaces.

[32] Gupta A., Sakthivel T., Seal S. (2015). Recent development in 2D materials beyond graphene. Prog. Mater. Sci..

[33] Khan S.I.U.A., Khan U., Ahmed N., Mohyud-Din S.T., Khan I., Nisar K.S. (2021). Thermal transport investigation in AA7072 and AA7075 aluminum alloys nanomaterials based radiative nanofluids by considering the multiple physical flow conditions. Sci. Rep..

[34] Hu T., Mei X., Wang Y., Weng X., Liang R., Wei M. (2019). Two-dimensional nanomaterials: Fascinating materials in biomedical field. Sci. Bull..

[35] Lu P., Liu Y., Zhou T., Wang Q., Li Y. (2018). Recent advances in layered double hydroxides (LDHs) as two-dimensional membrane materials for gas and liquid separations. J. Membr. Sci..

[36] Khorshidi B., Biswas I., Ghosh T., Thundat T., Sadrzadeh M. (2018). Robust fabrication of thin film polyamide-TiO_2_ nanocomposite membranes with enhanced thermal stability and anti-biofouling propensity. Sci. Rep..

[37] Vatanpour V., Paziresh S., Mehrabani S.A.N., Feizpoor S., Habibi-Yangjeh A., Koyuncu I. (2022). TiO_2_/CDs modified thin-film nanocomposite polyamide membrane for simultaneous enhancement of antifouling and chlorine-resistance performance. Desalination.

[38] Li D., Zhang J., Ahmed S.M., Suo G., Wang W., Feng L., Hou X., Yang Y., Ye X., Zhang L. (2020). Amorphous carbon coated SnO2 nanohseets on hard carbon hollow spheres to boost potassium storage with high surface capacitive contributions. J. Colloid Interface Sci..

[39] Liang B., Zhang P., Wang J., Qu J., Wang L., Wang X., Guan C., Pan K. (2016). Membranes with selective laminar nanochannels of modified reduced graphene oxide for water purification. Carbon.

[40] Sun P.-F., Yang Z., Song X., Lee J.H., Tang C.Y., Park H.-D. (2021). Interlayered Forward Osmosis Membranes with Ti3C2T x MXene and Carbon Nanotubes for Enhanced Municipal Wastewater Concentration. Environ. Sci. Technol..

[41] Sapkota B., Liang W.T., VahidMohammadi A., Karnik R., Noy A., Wanunu M. (2020). High permeability sub-nanometre sieve composite MoS_2_ membranes. Nat. Commun..

[42] Zhao D.L., Zhao Q., Chung T.-S. (2021). Fabrication of defect-free thin-film nanocomposite (TFN) membranes for reverse osmosis desalination. Desalination.

[43] Yang C., Li S., Lv X., Li H., Han L., Su B. (2021). Effectively regulating interfacial polymerization process via in-situ constructed 2D COFs interlayer for fabricating organic solvent nanofiltration membranes. J. Membr. Sci..

[44] Ang M.B.M.Y., Trilles C.A., De Guzman M.R., Pereira J.M., Aquino R.R., Huang S.-H., Hu C.-C., Lee K.-R., Lai J.-Y. (2019). Improved performance of thin-film nanocomposite nanofiltration membranes as induced by embedded polydopamine-coated silica nanoparticles. Sep. Purif. Technol..

[45] Ahmad N.A., Goh P.S., Wong K.C., Zulhairun A.K., Ismail A.F. (2020). Enhancing desalination performance of thin film composite membrane through layer by layer assembly of oppositely charged titania nanosheet. Desalination.

[46] Wang S., Yi Z., Zhao X., Zhou Y., Gao C. (2017). Aggregation suppressed thin film nanocomposite (TFN) membranes prepared with an in situ generation of TiO_2_ nanoadditives. RSC Adv..

[47] Wu X., Yang L., Meng F., Shao W., Liu X., Li M. (2021). ZIF-8-incorporated thin-film nanocomposite (TFN) nanofiltration membranes: Importance of particle deposition methods on structure and performance. J. Membr. Sci..

[48] Jiang C., Zhang L., Li P., Sun H., Hou Y., Niu Q.J. (2020). Ultrathin Film Composite Membranes Fabricated by Novel In Situ Free Interfacial Polymerization for Desalination. ACS Appl. Mater. Interfaces.

[49] Sui X., Yuan Z., Yu Y., Goh K., Chen Y. (2020). 2D Material Based Advanced Membranes for Separations in Organic Solvents. Small.

[50] Liu P., Hou J., Zhang Y., Li L., Lu X., Tang Z. (2020). Two-dimensional material membranes for critical separations. Inorg. Chem. Front..

[51] Liu G., Jin W., Xu N. (2016). Two-Dimensional-Material Membranes: A New Family of High-Performance Separation Membranes. Angew. Chem. Int. Ed..

[52] Sun H., Dong J., Liu F., Ding F. (2021). Etching of two-dimensional materials. Mater. Today.

[53] Buelke C., Alshami A., Casler J., Lin Y., Hickner M., Aljundi I.H. (2019). Evaluating graphene oxide and holey graphene oxide membrane performance for water purification. J. Membr. Sci..

[54] Nie L., Chuah C.Y., Bae T.H., Lee J.M. (2021). Graphene-Based Advanced Membrane Applications in Organic Solvent Nanofiltration. Adv. Funct. Mater..

[55] Boretti A., Al-Zubaidy S., Vaclavikova M., Al-Abri M., Castelletto S., Mikhalovsky S. (2018). Outlook for graphene-based desalination membranes. npj Clean Water.

[56] Shao F., Su X., Shen X., Ren S., Wang H., Yi Z., Xu C., Yu L., Dong L. (2021). Highly improved chlorine resistance of polyamide reverse membrane by grafting layers of graphene oxide. Sep. Purif. Technol..

[57] Makhetha T., Moutloali R. (2021). Incorporation of a novel Ag-Cu@ZIF-8@GO nanocomposite into polyethersulfone membrane for fouling and bacterial resistance. J. Membr. Sci..

[58] Chong J., Wang B., Li K. (2016). Graphene oxide membranes in fluid separations. Curr. Opin. Chem. Eng..

[59] Shi J., Wu W., Xia Y., Li Z., Li W. (2018). Confined interfacial polymerization of polyamide-graphene oxide composite membranes for water desalination. Desalination.

[60] Choi H.-G., Shah A.A., Nam S.-E., Park Y.-I., Park H. (2019). Thin-film composite membranes comprising ultrathin hydrophilic polydopamine interlayer with graphene oxide for forward osmosis. Desalination.

[61] Seah M.Q., Lau W.J., Goh P.S., Ismail A.F. (2022). Greener synthesis of functionalized-GO incorporated TFN NF membrane for potential recovery of saline water from salt/dye mixed solution. Desalination.

[62] Carey M., Barsoum M. (2021). MXene polymer nanocomposites: A review. Mater. Today Adv..

[63] Sun Y., Li Y. (2021). Potential environmental applications of MXenes: A critical review. Chemosphere.

[64] Gao L., Li C., Huang W., Mei S., Lin H., Ou Q., Zhang Y., Guo J., Zhang F., Xu S. (2020). MXene/Polymer Membranes: Synthesis, Properties, and Emerging Applications. Chem. Mater..

[65] Gong K., Zhou K., Qian X., Shi C., Yu B. (2021). MXene as emerging nanofillers for high-performance polymer composites: A review. Compos. Part B Eng..

[66] Liu G., Shen J., Liu Q., Liu G., Xiong J., Yang J., Jin W. (2018). Ultrathin two-dimensional MXene membrane for pervaporation desalination. J. Membr. Sci..

[67] Hao L., Zhang H., Wu X., Zhang J., Wang J., Li Y. (2017). Novel thin-film nanocomposite membranes filled with multi-functional Ti3C2Tx nanosheets for task-specific solvent transport. Compos. Part A Appl. Sci. Manuf..

[68] Wu X., Ding M., Xu H., Yang W., Zhang K., Tian H., Wang H., Xie Z. (2020). Scalable ti_3_c_2_t _x_ mxene interlayered forward osmosis membranes for enhanced water purification and organic solvent recovery. ACS Nano.

[69] Wen Y., Zhang X., Li X., Wang Z., Tang C.Y. (2020). Metal-Organic Framework Nanosheets for Thin-Film Composite Membranes with Enhanced Permeability and Selectivity. ACS Appl. Nano Mater..

[70] Dhanjai S.A., Tan B., Huang Y.J., Zhao H.M., Dang X.M., Chen J.P., Jain R. (2018). MoS_2_ nanostructures for electrochemical sensing of multidisciplinary targets: A review. Trac-Trends Anal. Chem..

[71] Ran J., Zhang P., Chu C., Cui P., Ai X., Pan T., Wu Y., Xu T. (2020). Ultrathin lamellar MoS_2_ membranes for organic solvent nanofiltration. J. Membr. Sci..

[72] Wang X., Liu Y., Fan K., Cheng P., Xia S. (2022). Nano-Striped Polyamide Membranes Enabled by Vacuum-Assisted Incorporation of Hierarchical Flower-Like Mos2 for Enhanced Nanofiltration Performance. SSRN.

[73] Wang Q., Wu X., Chen J., Li W., Zhang H., Wang J. (2020). Ultrathin and stable organic-inorganic lamellar composite membrane for high-performance organic solvent nanofiltration. Chem. Eng. Sci..

[74] Zhang H., Taymazov D., Li M.-P., Huang Z.-H., Liu W.-L., Zhang X., Ma X.-H., Xu Z.-L. (2019). Construction of MoS2 composite membranes on ceramic hollow fibers for efficient water desalination. J. Membr. Sci..

[75] Kadhom M., Deng B. (2018). Metal-organic frameworks (MOFs) in water filtration membranes for desalination and other applications. Appl. Mater. Today.

[76] Yang S., Wang Y., Lu P., Jin H., Pan F., Shi Z., Jiang X.-S., Chen C., Jiang Z., Li Y. (2020). Metal-Organic Frameworks Corset with a Thermosetting Polymer for Improved Molecular-Sieving Property of Mixed-Matrix Membranes. ACS Appl. Mater. Interfaces.

[77] Van Goethem C., Verbeke R., Hermans S., Bernstein R., Vankelecom I.F.J. (2016). Controlled positioning of MOFs in interfacially polymerized thin-film nanocomposites. J. Mater. Chem. A.

[78] Vaitsis C., Sourkouni G., Argirusis C. (2019). Metal Organic Frameworks (MOFs) and ultrasound: A review. Ultrason. Sonochemistry.

[79] Qian Y., Zhang F., Pang H. (2021). A Review of MOFs and Their Composites-Based Photocatalysts: Synthesis and Applications. Adv. Funct. Mater..

[80] Liu Y., Wang X.-P., Zong Z.-A., Lin R., Zhang X.-Y., Chen F.-S., Ding W.-D., Zhang L.-L., Meng X.-M., Hou J. (2022). Thin film nanocomposite membrane incorporated with 2D-MOF nanosheets for highly efficient reverse osmosis desalination. J. Membr. Sci..

[81] Górriz C.E., Zapata J.A., Etxeberría-Benavides M., Téllez C., Coronas J. (2019). Polyamide/MOF bilayered thin film composite hollow fiber membranes with tuned MOF thickness for water nanofiltration. Sep. Purif. Technol..

[82] Zhang X., Cheng F.-Y., Zhang H.-Z., Xu Z.-L., Xue S., Ma X.-H., Xu X.-R. (2020). In-situ synthetic modified metal-organic framework (MZIF-8) as an interlayer of the composite membranes for ethanol dehydration. J. Membr. Sci..

[83] Li G., Zhang K., Tsuru T. (2017). Two-Dimensional Covalent Organic Framework (COF) Membranes Fabricated via the Assembly of Exfoliated COF Nanosheets. ACS Appl. Mater. Interfaces.

[84] Sahoo R., Mondal S., Pal S.C., Mukherjee D., Das M.C. (2021). Covalent-Organic Frameworks (COFs) as Proton Conductors. Adv. Energy Mater..

[85] Shevate R., Shaffer D.L. (2022). Large-Area 2D Covalent Organic Framework Membranes with Tunable Single-Digit Nanopores for Predictable Mass Transport. ACS Nano.

[86] Fan H., Mundstock A., Feldhoff A., Knebel A., Gu J., Meng H., Caro J. (2018). Covalent Organic Framework-Covalent Organic Framework Bilayer Membranes for Highly Selective Gas Separation. J. Am. Chem. Soc..

[87] Chandra S., Chowdhury D.R., Addicoat M., Heine T., Paul A., Banerjee R. (2017). Molecular Level Control of the Capacitance of Two-Dimensional Covalent Organic Frameworks: Role of Hydrogen Bonding in Energy Storage Materials. Chem. Mater..

[88] Sun Q., Aguila B., Perman J., Nguyen N., Ma S. (2016). Flexibility Matters: Cooperative Active Sites in Covalent Organic Framework and Threaded Ionic Polymer. J. Am. Chem. Soc..

[89] He Y., Lin X., Chen J., Zhan H. (2021). Fabricating novel high-performance thin-film composite forward osmosis membrane with designed sulfonated covalent organic frameworks as interlayer. J. Membr. Sci..

[90] Yuan J., Wu M., Wu H., Liu Y., You X., Zhang R., Su Y., Yang H., Shen J., Jiang Z. (2019). Covalent organic framework-modulated interfacial polymerization for ultrathin desalination membranes. J. Mater. Chem. A.

[91] Jiang Y., Li S., Su J., Lv X., Liu S., Su B. (2021). Two dimensional COFs as ultra-thin interlayer to build TFN hollow fiber nanofiltration membrane for desalination and heavy metal wastewater treatment. J. Membr. Sci..

[92] Ji C., Zhai Z., Jiang C., Hu P., Zhao S., Xue S., Yang Z., He T., Niu Q.J. (2021). Recent advances in high-performance TFC membranes: A review of the functional interlayers. Desalination.

